# Green Separation by Using Nanofiltration of *Tristerix tetrandus* Fruits and Identification of Its Bioactive Molecules through MS/MS Spectrometry

**DOI:** 10.3390/plants13111521

**Published:** 2024-05-31

**Authors:** Nicolás Cifuentes-Araya, Mario Simirgiotis, Beatriz Sepúlveda, Carlos Areche

**Affiliations:** 1 Departamento de Química, Facultad de Ciencias, Universidad de Chile, Las Palmeras 3425, Nuñoa, Santiago 8320000, Chile; nicocifuentesaraya@gmail.com; 2 Instituto de Farmacia, Facultad de Ciencias, Campus Isla Teja, Universidad Austral de Chile, Valdivia 5090000, Chile; mario.simirgiotis@uach.cl; 3 Departamento de Ciencias Químicas, Universidad Andrés Bello, Campus Viña del Mar, Quillota 980, Viña del Mar 2520000, Chile

**Keywords:** nanofiltration, *Tristerix tetrandus*, phenolics, amino acids, plant-derived food, new plant products

## Abstract

Membrane technology allows the separation of active compounds, providing an alternative to conventional methods such as column chromatography, liquid–liquid extraction, and solid–liquid extraction. The nanofiltration of a Muérdago (*Tristerix tetrandus* Mart.) fruit juice was realized to recover valuable metabolites using three different membranes (DL, NFW, and NDX (molecular weight cut-offs (MWCOs): 150~300, 300~500, and 500~700 Da, respectively)). The metabolites were identified by ESI-MS/MS. The results showed that the target compounds were effectively fractionated according to their different molecular weights (MWs). The tested membranes showed retention percentages (RPs) of up to 100% for several phenolics. However, lower RPs appeared in the case of coumaric acid (84.51 ± 6.43% (DL), 2.64 ± 2.21% (NFW), 51.95 ± 1.23% (NDX)) and some other phenolics. The RPs observed for the phenolics cryptochlorogenic acid and chlorogenic acid were 99.74 ± 0.21 and 99.91 ± 0.01% (DL membrane), 96.85 ± 0.83 and 99.20 ± 0.05% (NFW membrane), and 92.98 ± 2.34 and 98.65 ± 0.00% (NDX membrane), respectively. The phenolic quantification was realized by UHPLC-ESI-MS/MS. The DL membrane allowed the permeation of amino acids with the MW range of about 300~100 Da (aspartic acid, proline, tryptophan). This membrane allowed the highest permeate flux (22.10–27.73 L/m^2^h), followed by the membranes NDX (16.44–20.82 L/m^2^h) and NFW (12.40–14.45 L/m^2^h). Moreover, the DL membrane allowed the highest recovery of total compounds in the permeate during the concentration process (19.33%), followed by the membranes NFW (16.28%) and NDX (14.02%). Permeate fractions containing phenolics and amino acids were identified in the membrane permeates DL (10 metabolites identified), NFW (13 metabolites identified), and NDX (10 metabolites identified). Particularly, tryptophan was identified only in the DL permeate fractions obtained. Leucine and isoleucine were identified only in the NFW permeate fractions, whereas methionine and arginine were identified only in the NDX ones. Liquid permeates of great interest to the food and pharmaceutical industries were obtained from plant resources and are suitable for future process optimization and scale-up.

## 1. Introduction

Muérdago (*Tristerix tetrandus*) is a medicinal mistletoe species native to southern Argentina and central and southern Chile. This plant is a parasite of aspen (*Populus* sp.), colliguay (*Colliguaya odorifera*), maqui (*Aristotelia chilensis*), willow (*Salix* sp.), among other native Chilean species. It is commonly gathered by local collectors, dried, and sold in local markets. This plant has traditionally been used in alternative medicine as an anti-inflammatory, digestive [1,2], hemostatic, and hypocholesterolemic [3] remedy and as an anxiolytic agent [4]. *Tristerix tetrandus* contains a wide and important number of phenolics and anthocyanins in its fruits and leaves [1,2], and some other mistletoe plants have shown the presence of several amino acids [5].

Several phenolics are bioactive compounds [6], and they are widely distributed in fruits and vegetables, such as blueberries, blackberries, spinach, among others [7,8], and have the ability to protect against several human diseases [8,9,10]. Phenolics such as quinic acid, rutin, quercetin, caffeoyl-glucose, p-coumaric acid, catechin, 5-O-caffeoylquinic acid (3-CQA), among others are contained in *Tristerix tetrandus* [1]. This plant possesses high concentrations of phenolic compounds, which have been found in studies in vitro and in vivo to possess a range of biological activities including anticancer and anti-platelet activities, as well as antioxidant properties [11]. In addition, several amino acids have been found in several mistletoe species [12]. Amino acids have regulatory roles in cell metabolism and function [13]. Indispensable amino acids contained in food are needed to synthesize bodily proteins [14].

The extraction of natural products, such as phenolics, is traditionally realized by using conventional procedures, including toxic organic solvents [15], but the solvent extractions do not ensure that the liquid fraction obtained contains specific phenolic molecules according to their molecular size and/or molecular charge. Moreover, the driving force for conventional extraction methods (e.g., maceration, hydro distillation, water distillation, and steam distillation) is the application of heat mixing as well as toxic solvents. The problem with these methods is that they are expensive, time-consuming, have low extraction selectivity, cause thermal degradation of thermolabile compounds, among others. On the other hand, metabolites such as amino acids are hydrophilic and are, therefore, difficult compounds for conventional solvent extraction [16]. Thus, there arises the need to study novel, effective, and green techniques for the extraction of bioactive compounds. The alternative extraction and isolation of bioactive molecules would find beneficial and specific applications in the food, pharmaceutical, and phytochemical industries.

Membrane technology has allowed the separation of bioactive compounds from a wide variety of plant and food solutions [17]. High recovery or removal efficiency, low energy input, environmental safety, high selectivity, easy scale-up, low temperature processing, absence of phase transition, and versatile integration with other unit operations make membrane fractionation an appropriate technology for the treatment of organic and thermolabile solutions. The use of nanofiltration (NF) is an advantageous, green, and clean alternative for the purification of natural compounds by selecting membranes with a suitable molecular weight cut-off (MWCO) (150–1000 Da range) [7,18] and according to the target metabolites that are present in the treated solutions. Therefore, the application of NF appears as a novel alternative in the field of natural products to the conventionally used methods for the isolation, fractionation, and identification of pure compounds.

The separation and characterization of bioactive molecules contained in native plants are important for the preparation of nutraceuticals and food ingredients. Liquid chromatography (HPLC, UPLC, UHPLC) coupled to several mass spectrometers such as flight time (TOF or Q-TOF), quadrupole-orbitrap (Q or Q-OT), triple quadrupole (TQ), or quadrupole-electrospray ionization (Q-ESI) for metabolomic profiling and biological analysis in dietary supplements, plants, fruits, and vegetables has increased over the last few years [1].

Until now, no information regarding the NF of Muérdago fruit juice and the isolation of its bioactive compounds is available in the literature. Muérdago fruit juice contains valuable metabolites, which can be selectively fractionated through NF and allow the creation of specific fractions for use on food. In this way, the aim of this work was to evaluate the NF of a Muérdago fruit juice and to identify the fractionated metabolites (phenolics and amino acids) by using ESI-MS/MS. Specifically, three NF membranes were used, sequentially and separately, starting from the membrane with the smallest pore size and ending up by using the membrane with the biggest pore size. The process performance evolution, the fouling formation, a chemical cleaning procedure, and the specific molecule fractionation were evaluated. ESI-MS/MS analysis was used to identify several metabolites and bioactive compounds contained in the Muérdago fruit, the quantification of some phenolics, and their observation and evaluation during NF.

## 2. Results and Discussion

### 2.1. Visual Membrane Inspection and Characterization

**Figure 1:**
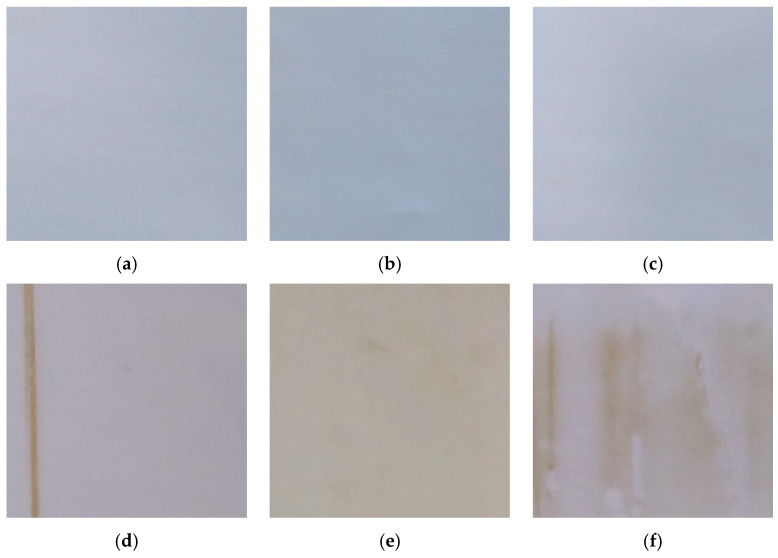
Membrane cuts (DL, NFW, NDX) used during the treatments: (**a**) Original DL membrane; active surface), (**b**) original NFW membrane; active surface, (**c**) original NDX membrane; active surface, (**d**) Muérdago solution; 10 mg/L; used DL active surface; 10-bar TMP, (**e**) Muérdago solution; 10 mg/L; used NFW active surface; 30-bar TMP, and (**f**) Muérdago solution; 10 mg/L; used NDX active surface; 25-bar TMP.

On the active layers of the original and of the used membranes, digital camera photographs were taken to identify and to characterize the aspect of the original membrane material and to compare it with that of the tested membranes and the presence of fouling layers on each of them (Figure 1). These photographs were taken only to inspect the mentioned membrane surfaces visually.

Figure 1a–c show the active surfaces of the original DL, NFW, and NDX membranes, respectively. They presented a quite clear and clean surface before any treatment was realized. Figure 1d–f present the active surfaces of the used DL, NFW, and NDX membranes. Fouling layers were observed on all of them. The fouling layers formed became more important as the MWCO of the used membrane was bigger. This way, the DL membrane presented the less important fouling layer among the membranes tested. On all the treated membranes, the fouling had the appearance of an organic layer, not very solid, and of a yellow-oxide color, becoming somewhat darker in the more fouled zones. Particularly, the active layer of the used NFW membrane presented a completely yellow surface. It would have been majorly formed by phenolics and probably by some anthocyanins present in the Muérdago fruit [1]. Fouling layers formed by these compounds have been observed during the nanofiltration of blueberry aqueous extracts (anthocyanin fouling) [19], the nanofiltration of model juice solutions (phenolics) [20], among others.

### 2.2. Membrane Process Parameters

#### 2.2.1. Permeate Flux

Table 1 presents the permeate flux observed during each membrane treatment.

Table 1 shows the permeate flux values observed at the processing times 5 min and 180 min during each membrane treatment. The two-way RM ANOVA indicated that the permeate flux decreased significantly during the DL treatment (between the processing times 5 and 180 min). This permeate flux decay would have been caused by the formation of fouling described visually (Figure 1d), acting as an additional barrier. Differently, during the treatments NFW and NDX, the permeate flux underwent a significant increase between the processing times 5 and 180 min. The increase observed during the treatment NFW was less important than that observed during the treatment NDX (Table 1). Increments in the permeate flux have been described during the nanofiltration of solutions containing Cu and Cr [21], during the nanofiltration of whey [22], and also during the nanofiltration of phenolics [23], all of them due to an increase in the feed temperature. In the present study, the temperature increased from 22.1 ± 0.01 °C up to 29.1 ± 0.01 °C during the DL treatment, from 22.8 ± 0.01 °C up to 28.5 ± 0.01 °C during the NFW treatment, and from 22.4 ± 0.01 °C up to 28.1 22.4 ± 0.01 °C during the NDX treatment. The flux increment would have been due in part to the temperature increase observed in the feed solutions, no matter the membrane treatment effected (NFW or NDX), and to the high TMP applied. Further, the influence of the surfacial chemical properties of the formed fouling layers would have also led to increase the permeate flux through the tested membranes NFW and NDX. Significant fouling layers were visually observed on the active layers of these mentioned membranes (Figure 1e,f).

#### 2.2.2. Membrane Filtration Assessment

The results related to the filtration performance of the NF membranes DL, NFW, and NDX before (new material) and after the cleaning procedure realized (used membranes) are here presented. Figure S1 (Supplementary Materials) displays the average “permeate flux versus transmembrane pressure (TMP)” curves worked out while filtering distilled water through the new membrane material (DL, NFW, and NDX). These curves show that the permeate flux observed through the DL membrane was quite higher than those observed through the NFW and the NDX membranes, respectively, at the same TMP applied (5, 10, 15, 20, and 25 bar). Further, it was observed that the curves obtained for the cleaned membranes, respectively, did not differ from those observed for the new membrane material. This indicates that the membrane fouling observed was not irreversible and that the original membrane integrity can be recovered through chemical cleaning after the filtration of the tested Muérdago juice. The decrease in the permeate flux during the DL treatments (Table 1) shows the way the fouling layers acted on the membrane performance. The increases in the permeate flux during the NFW and NDX treatments show how this parameter varied according to the fouling layers formed and the processing conditions applied.

#### 2.2.3. Membrane Resistance (MR)

The membrane resistance values measured on the new membranes and on the washed membranes are presented in Table 2. The variation in the hydraulic MR indicates the membrane integrity, its stability, and specific performance. These values were calculated using Equation (1).

The two-way ANOVA detected significant differences in the MR due to the different membranes tested (DL, NFW, and NDX) (*p* < 0.001), but not due to the different membrane states (new and washed). The new DL membrane presented the lowest MR among the treated new membranes, whereas the new NFW and NDX membranes showed significantly higher MR values (*p* < 0.001), and both were close to each other in magnitude. It occurred similarly in the case of the washed DL, NFW, and NDX membranes. Generally, the DL membrane presented significantly lower MR values despite its lower MWCO in relation to the membranes NFW and NDX. No significant differences were observed between the new and the washed material in the particular case of the membranes DL, NFW, and NDX. The lower MR was related to the membrane (DL), and allowing this way the highest permeate flux among the membranes tested. At similar TMP values, this membrane would allow a more important permeation of solids through it than the NFW and NDX membranes.

#### 2.2.4. Feed pH and Electrical Conductivity

**Figure 2:**
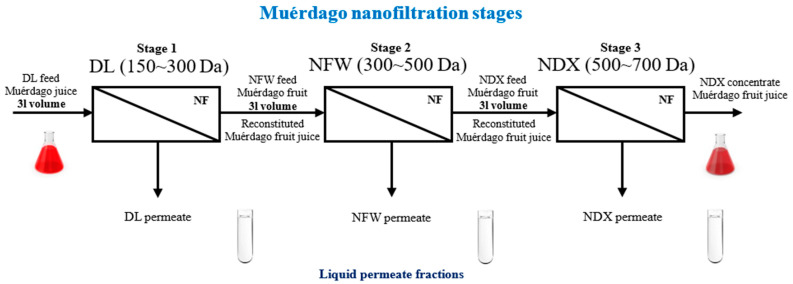
Scheme of the sequential NF stages carried out on the Muérdago fruit.

Table 3 shows the processing parameters measured (EC, pH) in the feed streams during each separate membrane treatment effected. These parameters contribute to the understanding of each of the three NF stages realized (Figure 2). The feed pH value reveals to some extent the migration of acidic and/or alkaline compounds through each tested membrane.

The two-way RM ANOVA detected significant differences in the feed EC values according to the different treatments carried out (*p* = 0.009) and to the different processing times considered (*p* < 0.001) (Table 3). A significant interaction was found for the factors “membraneXprocessing-time” (*p* = 0.012). The EC increased significantly in all cases (treatments DL, NFW, and NDX (Figure 2)) during the processing from the time 0 min up to the time 180 min, but it increased more significantly during the DL treatment (Table 3). The higher permeate flux observed (Table 1) would have allowed a more important water depletion through the DL membrane, increasing the feed EC in higher magnitude. Indeed, significant differences were found among the EC values observed during the DL treatments and those observed during the NFW and NDX treatments at each particular processing time evaluated. However, no significant differences were observed between the membranes NFW and NDX at each particular processing time. This denotes the significant influence of the permeate flux on the EC observed, since the temperature variation during the three membrane treatments was similar.

Significant differences appeared in the feed pH value according to the different membranes used (*p* < 0.001) and also to the different processing times during each consecutive treatment (*p* < 0.001). The pH value decreased significantly during all the treatments carried out, showing a slightly more important decrease during the NFW treatment (Table 3). A decrease in the feed pH denotes the concentration of acidic compounds in the feed stream thanks to their retention by the membrane, coupled to a continuous water permeation. This feed pH decrease, together with an increase in the feed EC, indicates a consistent permeate stream occurring through the tested membranes (DL, NFW, and NDX), composed of water and certain depleted molecules. The DL membrane allowed a higher permeate flux (Table 1) than the two other ones (NFW and NDX) at a lower TMP, but the associated feed pH variation was the lowest one. This suggests that more acidic species were allowed to permeate this membrane in comparison with those that permeated the NFW and the NDX membranes, respectively. Along the three membrane treatments realized, the feed pH value kept itself in an acceptable value, regarding a fresh fruit feed solution. Globally, the feed pH value decreased significantly and continuously from the beginning of treatment DL up to the end of treatment NDX (Table 3). This denotes that the pH value decreased continuously mainly because of the concentration of acidic species in the feed streams and not due to the slight variation observed in the feed temperature (similar during the three membrane treatments). The exposed results and the variations observed in the EC and in the feed pH during each respective membrane treatment show that the permeate flux is different in magnitude through each tested membrane (Table 1), but in all cases, it is composed of solids and majorly of water.

### 2.3. UHPLC-MS Analysis

Over the samples taken from the feed and from the permeate streams along the processing, UHPLC-MS analysis was carried out to identify and to quantify the presence of phenolics.

#### 2.3.1. Metabolomic Profiling Using UHPLC-ESI-MS/MS and Tentative Identification

Samples of the processed liquid extract (Muérdago, *Tristerix tetrandus* Mart.) (from the feed and permeate streams) obtained at the processing times 5 and 180 min, respectively, were filtered and injected in the UHPLC-ESI-MS/MS equipment. Phenolics and amino acids were identified tentatively in these samples.

##### Phenolics

The UHPLC-MS analysis carried out allowed the identification of several phenolics contained in the Muérdago liquid extract, in the feed, and in the permeate samples. These molecules were found tentatively during each of the different membrane treatments effected (DL, NFW, NDX). The molecules identified in the Muérdago fruit are presented in Table S2 (Supplementary Materials), and their peaks were identified and assigned with [M − H]^−^ and [M + H]^+^ ions to each respective theoretical mass (*m/z*). The phenolics quinic acid (C_7_H_12_O_6_), 3-O-caffeoylquinic acid (3-CQA) (C_15_H_18_O_9_), p-coumaroyl malate (C_13_H_12_O_7_), isorhamnetin (C_16_H_12_O_7_), apigenin (C_10_H_10_O_5_), ferulic acid (C_10_H_10_O_4_), ellagic acid (C_15_H_10_O_5_), 7-O-Methylisorhamnetin (C_17_H_14_O_7_), and chrysin (C_15_H_10_O_4_) were tentatively identified. The phenolics gallic acid (C_7_H_6_O_5_), cryptochlorogenic acid (C_16_H_18_O_9_), chlorogenic acid (C_16_H_18_O_9_), caffeic acid (C_9_H_8_O_4_), p-coumaric acid (C_9_H_8_O_3_), rutin (C_15_H_10_O_8_), and quercetin (C_15_H_10_O_7_) were directly identified and quantified by UHPLC-ESI-MS/MS, using standards (see Table S2 in Supplementary Materials). A total of 16 phenolic compounds were identified in the Muérdago fruit [1,24]. Their respective presences in the feed and in the permeate samples are indicated in Table S2. Figure S2 (Supplementary Materials) displays schematically the three stages of fractionation process carried out consecutively, one after the other. It shows the presence of the mentioned phenolics during each different membrane treatment (DL, NFW, and NDX) and in each of the evaluated separation streams (feed and permeate).

##### Amino Acids

The UHPLC-MS analysis allowed the tentative identification of eight amino acids: aspartic acid (C_4_H_7_NO_4_), proline (C_5_H_9_NO_2_), valine (C_5_H_11_NO_2_), tryptophan (C_11_H_12_N_2_O_2_), leucine (C_6_H_13_NO_2_), isoleucine (C_6_H_13_NO_2_), methionine (C_5_H_11_NO_2_S), and arginine (C_6_H_14_N_4_O_2_) (Table S2, Supplementary Materials) [12,25,26].

### 2.4. Quantification of Phenolics through UHPLC-MS Analysis

The UHPLC-MS analysis allowed the quantification of seven phenolics: gallic acid (C_7_H_6_O_5_), cryptochlorogenic acid (C_16_H_18_O_9_), chlorogenic acid (C_16_H_18_O_9_), caffeic acid (C_9_H_8_O_4_), p-coumaric acid (C_9_H_8_O_3_), rutin (C_15_H_10_O_8_), and quercetin (C_15_H_10_O_7_). These phenolics were directly identified and quantified by the use of UHPLC-MS using standards. The respective concentrations measured in the feed and permeate samples are shown in Table 4.

Table 4 shows that the phenolics gallic acid, caffeic acid, rutin, and quercetin did not cross any of the tested membranes, remaining in the feed streams during the three treatments realized (Table S2, Figure S2, Supplementary Materials). Particularly, the concentration of caffeic acid found in the feed samples increased significantly during the consecutive membrane treatments (Table 4) despite the reconstitution of the treated solutions with distilled water at the end of the treatments DL and NFW (see Materials and Methods section). During the treatments effected, the remotion of other compounds through each membrane and the continuous water reconstitution after each treatment would have allowed a better quantification of caffeic acid in solution.

It occurred differently in the case of quercetin. Its concentration in the feed stream decreased as the feed solution (Table 4) was continuously and sequentially fractionated by the DL, NFW, and NDX treatments (Figure 2 and Figure S2, Supplementary Materials). Since no quercetin was found in the respective permeate samples, it seems that this phenolic participated in the formation of membrane fouling due to the color of the fouling layers observed (Figure 1d–f) and its mentioned decrease in the feed stream. The concentration of rutin showed a decrease between the feed samples DL and NFW. This molecule would have also participated in membrane fouling formation (Figure 1d–f). Rutin [27] and quercetin [28,29] precipitate as yellowish compounds.

The phenolic p-coumaric acid showed a particular behavior during the three NF stages. Its concentration increased significantly in the NFW permeate samples in comparison with the DL permeate samples. Hence, its permeation was more favored through the NFW membrane. The permeation of p-coumaric acid was also favored through the NDX if compared with the permeation observed through the DL membrane. Nevertheless, the concentration found in the NDX permeates was lower than that observed in the NFW ones (Table 4). The MW of p-coumaric acid (164.16 Da) allowed its permeation through the three tested membranes, but more importantly through the NFW membrane. Caffeic acid was retained in the feed stream during all the treatments carried out despite the fact that its MW (180.16 Da) should have favored its permeation through the three tested membranes (Table S1). A similar phenomenon was observed in the case of gallic acid (MW: 170.12 Da). During the membrane treatments effected, the membrane surfacial charge would have been negative, and even more negative during the DL treatments, according to the feed pH observed (Table 3). According to the gallic acid pKa (Table S2, Supplementary Materials), the phenolic charge would have been majorly negative. This membrane surfacial condition along with membrane fouling (acting as an additional permeation barrier) would have helped the membrane retention of gallic acid. A similar phenomenon would have occurred in the case of caffeic acid and during the NFW and NDX treatments for the phenolics rutin and quercetin. These compounds were not permeated through the membrane DL (Figure S2, Supplementary Materials) due to its small MWCO (150~300 Da) (Table S1) and to the MWs of rutin and quercetin (302.23 Da) (Table S2).

The phenolics chlorogenic acid and cryptochlorogenic acid showed the highest concentrations (ppb) among the quantified phenolics in the Muérdago fruit. Cryptochlorogenic acid was the most abundant phenolic in terms of concentration (Table 4). In the case of the phenolics that crossed in part the membranes tested (cryptochlorogenic acid, chlorogenic acid, and p-coumaric acid) (Figure S2, Supplementary Materials), it was seen that the permeate concentration was always quite lower than the respective feed one, except in the case of p-coumaric acid permeating the NFW membrane (Table 4). Particularly, the concentration of cryptochlorogenic acid remained stable in the feed stream through the consecutive membrane treatments (DL, NFW, NDX). In parallel, the concentration of chlorogenic acid increased between the treatments DL and NFW to then remain stable up to the treatment NDX. The DL membrane allowed the higher permeate flux observed (Table 1), which was in accordance with the lowest membrane resistance determined (Table 2). It was seen that important amounts of compounds were removed from the DL feed stream (Figure S2, Supplementary Materials) and allowed for a better quantification of chlorogenic acid in the NFW and NDX feed streams by UHPLC-MS/MS. The feed pH decays during the processing were due to the remotion of acidic molecules through the tested membranes. The pH value decreased in less magnitude through the DL membrane, and more amounts of acid molecules seem to have permeated the NFW and NDX membranes, while more alkaline compounds would have been retained in the feed streams.

#### 2.4.1. Retention Percentage (RP)

The three different membrane treatments realized led to the observation of different RPs for each phenolic quantified. Table 5 shows the RPs observed during each treatment effected. The RPs were calculated taking into account the concentrations of each compound quantified by UHPLC-MS, in the feed, and in the permeate samples (Equation (3)) for each specific membrane treatment.

The phenolics gallic acid, caffeic acid, rutin, and quercetin showed RPs of 100.00 ± 0.00% during all the treatments realized, being completely retained by each of the tested membranes, in each respective feed stream (Figure S2, Supplementary Materials). In turn, the phenolics cryptochlorogenic acid, chlorogenic acid, and p-coumaric acid were able to permeate in part the three treated membranes (Table 5, Figure S2, Supplementary Materials). Among these latter phenolics, cryptochlorogenic acid and chlorogenic acid were the two most retained molecules. Comparing the three treated membranes (DL, NFW, NDX), these two mentioned phenolics presented RPs between 99.74 ± 0.21% and 92.98 ± 2.34%, and between 99.91 ± 0.015 and 98.65 ± 0.00%, respectively (Table 5). Particularly, the phenolic p-coumaric acid showed an important decrease in its RP when comparing the membranes DL with NFW (84.51 ± 6.43% versus 2.64 ± 2.21%) (Table 5), but it increased again when comparing the membranes NFW with NDX (2.68 ± 2.21% versus 51.95 ± 1.23%) (Table 5). The molecular weight (MW) of this molecule is 164.16 Da, and it was allowed to cross the mentioned membranes (see Table S1 and Figure S2 in Supplementary Materials). Further, according to its pKa_1_ = 4.34 and pKa_2_ = 8.83 values, the p-coumaric acid molecule was positively charged during the membrane treatments. This positive charge increased from the DL treatments up to the NDX treatments according to the respective decreases in the pH values observed (Table 3). Moreover, at the feed pH values observed during the treatments, the membrane surfacial charge would have been negative since most polyamide-TFC membranes present isoelectric points in the range of pH 3~4 [30]. These conditions allowed the p-coumaric acid molecule to cross the mentioned membranes (Figure S2, Supplementary Materials).

In addition, it was observed that caffeic acid was completely retained by the membranes DL, NFW, and NDX (Figure S2, Supplementary Materials), despite its molecular weight (180.16 Da) being similar to that of p-coumaric acid (Table S2). The caffeic acid molecule possesses three hydroxyl groups, which would have conferred it a more important negative charge than the p-coumaric acid one since this last molecule has only two hydroxyl groups.

In parallel, the fouling layers visually observed on the used DL, NFW, and NDX membranes (Figure 1d–f) would have helped to reduce the membrane negative charge, retaining in part the p-coumaric acid molecule, despite its favored permeation through the tested membranes (Figure S2, Supplementary Materials). In comparison with the NFW treatments, the NDX increased the p-coumaric RP apparently due to less membrane fouling (Figure 2). Quercetin was retained effectively by all the membranes tested (100 ± 0.00%). Its higher molecular weight (302.40 Da) along with the presence of fouling were the responsible factors for its retention. The quercetin molecule during the processing would have been more positively charged (Table S2, Supplementary Materials), while the membrane surfacial charges would have been negative. The presence of fouling (visual appreciation) would have helped to retain the permeation of quercetin through the membranes.

The present study shows the feasibility of retaining some of the phenolics contained in the Muérdago fruit juice, while the phenolic p-coumaric acid is allowed to permeate the tested membranes, creating valuable permeate fractions (Figure S2, Supplementary Materials). p-coumaric acid is a phenolic that possesses interesting bioactive properties such as antioxidant, antimicrobial, anticancer, anti-arthritic, anti-inflammatory, gout prevention, anti-diabetic, anti-melanogenic, skin regeneration, gastroprotective, anti-ulcer, cardioprotective, hepatoprotective, reno-protective, bone formation, anti-angiogenic, anti-platelet, among others [31].

#### 2.4.2. Bioactive Compound Fractionation

The UHPLC-MS analysis allowed the tentative identification of several phenolics and amino acids contained in the liquid Muérdago juice treated by membranes, in the feed, and in the permeate samples. Concerning the fractionation of amino acids, it was observed that aspartic acid was consecutively present in almost all the samples taken, but not in the sample P-NDX(t180) (Table S2). Its passage was allowed by all the membranes tested (Figure S2, Supplementary Materials), but the treated solution ran out of aspartic acid during the NDX treatment, being absent in the NDX permeate stream. The presence of both methionine and arginine was detected only in the permeate streams during the NDX treatments (Figure S2, Supplementary Materials). Their presence would have been masked by the rest of the compounds present in the feed solution during the previous treatments (DL and NFW) when the feed solutions were more concentrated in total compounds. It occurred similarly in the case of leucine and isoleucine, but instead, these amino acids were identified only in the NFW permeate samples (Table S2, Figure S2, Supplementary Materials). In the DL permeate, the amino acid tryptophan was detected, which did not appear in the permeates NFW and NDX (Table S2, Figure S2, Supplementary Materials). It was observed that the amino acids aspartic acid and proline were present in the permeate samples DL and NFW, whereas valine was identified in the samples F-NFW(t5), P-NFW(t180), F-NDX(t5), and P-NDX(t180) (Table S2, Supplementary Materials). This amino acid appeared only in the NFW and NDX permeates. All the mentioned amino acids were detected in the permeates due to their allowed membrane permeation and thanks to the membrane retention of the majority of the compounds present in the feed solution. It was seen that all the identified amino acids were found in the permeate samples, but the sample P-NFW contained greater amounts of them (aspartic acid, proline, valine, leucine, isoleucine) (Table S2). The permeate sample P-DL contained the amino acids aspartic acid, proline, and tryptophan, whereas the sample P-NDX contained the amino acids valine, methionine, and arginine. A total of eight amino acids were tentatively identified during the membrane treatments.

Concerning the phenolics found tentatively, quinic acid and 3-O-caffeoylquinic acid (3-CQA) were allowed to cross the DL membrane and then the NFW membrane (Table S2, Supplementary Materials). Surprisingly, the phenolic 3-CQA (MW: 354.31 Da) was able to permeate the DL membrane despite its MWCO (150~300 Da) (Table S1, Supplementary Materials). During the DL treatments, the molecular charge of 3-CQA would have been closer to neutrality, and thanks to the high TMP values applied, it crossed in part the tested membranes. The phenolics 3-CQA and quinic acid are considered beneficial to human health [32,33], and their presence in the mentioned permeates creates an additional value. Through the continuous fractionation realized, the concentration of quinic acid would not have been significant enough to appear in the P-NDX(t180) samples, as was the case of 3-CQA (Table S2).

Ellagic acid was a phenolic retained effectively during the three effected membrane treatments. Its retention by the DL membrane is explained by its MW and the MWCO of the DL membrane. Further, according to its pKa values (Table S2), its molecular charge during the treatments would have been positive. Ellagic acid was retained by the membranes NFW and NDX, despite the phenolic MW and the respective MWCO of the mentioned membranes. The fouling layers observed (Figure 1e,f) would have acted as an additional barrier restricting the permeation of ellagic acid through them and modifying the surfacial membrane properties.

Ferulic acid appeared only in the F-NFW(t5), P-NFW (t180), and P-NDX (t180) samples. This molecule would have crossed all the membranes tested thanks to its MW (194.18 Da) and pKa values (Table S2). The molecule charge was not far from its isoelectric point during the DL treatments (Table S2), and progressively, it became more positive during the NFW and NDX treatments (Table 3). This way, it was allowed to permeate due to the high TMP imparted. The fractionation process was ruled by the molecular sizes of the identified bioactive compounds and by the intrinsic characteristic of the tested NF membranes. Permeate fractions containing ferulic acid were identified during the NFW and NDX treatments. This phenolic possesses important properties to improve human health [11].

#### 2.4.3. Permeation Percentage (PP) of Total Solids during the NF of Muérdago Fruit Juice

The PP of total solids is presented in Table 6 for each membrane treatment realized. It points out the recovery of total solid compounds contained in the permeate during the concentration process.

According to Table 6 and to the two-way ANOVA, the PP was higher during the DL treatment in comparison to the NFW and to the NDX treatments (*p* < 0.0010), starting from a PP of 20.63 ± 0.13% (DL(t5)), and decreasing significantly (*p* < 0.0010) to 18.02 ± 0.18% (DL(t180)). Differently, the PP increased significantly (*p* < 0.0010) during the NDX treatment, from 12.54 ± 0.12% up to 15.49 ± 0.08%, and also during the NFW treatment (*p* < 0.0010), from 9.89 ± 0.13% up to 11.13 ± 0.11%. This increase in the PP would be due to the continuous fouling formed (Figure 1d) during the mentioned treatments and the respective increments observed in the permeate flux (Table 1). The intrinsic characteristics of the fouling layer visually observed would have favored the passage of some solids through it, as was observed in the case of the phenolic p-coumaric acid (Table 4) during the NFW and NDX treatments. Differently, the decrease observed in the PP during the DL treatments would be explained by the fouling formed (Figure 1d–f), which would have acted in this case as an additional barrier against the permeation of molecules through the membranes. In parallel, the permeate flux observed underwent a low but significant decrease (Table 1).

The PPs observed indicate that considerable amounts of metabolites and bioactive compounds permeated the tested membranes. This way, valuable permeated fractions enriched in these compounds were obtained. Permeate fractions of great interest to the food and pharmaceutical industries were created and were suitable for process optimization scale-up. The DL membrane presented an interesting and practical behavior since the permeate flux was the highest one, and the less important fouling amounts were observed. The NFW membrane presented an interesting behavior due to the more favored permeation of p-coumaric acid and the more important number of amino acids found in the respective permeate. p-coumaric acid has been found to possess different bioactive properties such as antioxidant, antimicrobial, anticancer, anti-arthritic, anti-inflammatory, gout prevention, anti-diabetic, anti-melanogenic, skin regeneration, gastroprotective, anti-ulcer, cardioprotective, hepatoprotective, reno-protective, etc. [31]. The NDX permeates obtained were interesting liquid fractions since they contained the particular presence of arginine and methionine.

## 3. Materials and Methods

### 3.1. Muérdago Extract Solution Preparation

Muérdago (Chilean mistletoe, *Tristerix tetrandus*) fruits collected in southern Chile were freeze-dried and stored at room temperature in complete darkness. Then, the freeze-dried fruits were milled into finely ground flour using a laboratory grinding machine (Polymix^®^ PX-MFC 90D, Kinematica AG, Malters, Switzerland), at 220 rpm, and stored hermetically in a freezer at −20 °C until used. Then, 10 g of the finely ground Muérdago flour was dissolved into three liters of distilled water (electrical conductivity (EC) < 4 µS/cm (pH = 6.5 ± 0.2)) during one hour at room temperature. Immediately afterwards, the Muérdago solution prepared was filtered twice through cotton with the aims of removing all the possible pectin material contained in it and protecting the NF membrane integrity. Then, the resulting solution was filtered twice, using two layers of gauze as a first step, and then subjected to three consecutive ultrasound baths (Ultrasonic TI-H 20; Elma Schmidbauer GmbH, Singen, Germany), treated with an ultrasonic power of 100% (250 W) under an ultrasonic frequency of 35 kHz for a period of 15 min each. After the ultrasound baths, the solution was newly filtered through filter paper under vacuum. This last procedure was repeated three times, until no more accumulated matter was observed on the filter paper material. The resulting liquid extract was reconstituted up to three liters using distilled water and immediately used as the feed solution for the membrane fractionation process. This last reconstitution was realized to obtain the solution volume lost during the previous filtrations. Muérdago fruit was chosen for the membrane treatments since its extracts possess a wide variety of interesting bioactive molecules, which molecular weights range from approximately 100 Da up to 700 Da, considering amino acids [12] and phenolics. These molecules were the target fractionation materials.

### 3.2. Membrane Materials

Three different polyamide-TFC NF membranes with different molecular weight cut-offs (MWCOs) were used, which were purchased from Sterlitech Corporation, Auburn, WA, USA. The membranes were a DL membrane (Suez (GE)^TM^) (pore size/MWCO: 150~300 Da), an NFW membrane (Synder^TM^) (pore size/MWCO: 300~500 Da), and an NDX membrane (Synder^TM^) (pore size/MWCO: 500~700 Da). Table S1 (Supplementary Materials) displays the technical specifications of the mentioned membrane materials. The three membranes were selected according to the target bioactive molecules contained in the Muérdago fruit juice and the membranes’ MWCOs (Table S1), which should be able to separate the treated biomolecules into different and profitable liquid fractions.

### 3.3. Protocol

The Muérdago fruit juice was fractionated using a crossflow membrane filtration system (CF042D membrane separation cell (Delrin Acetal)) (Sterlitech Corporation, Auburn, WA, USA). A Hydracell M03-S pump (positive displacement, diaphragm pump (Wanner Engineering, Minneapolis, MN, USA)) was used as a feed-flow pump to operate the CF042D crossflow cell unit and to pump the treated fluid through the entire fractionation system. The NF membranes were carefully cut and disposed into the membrane module, which had a 42 cm^2^ effective area. Three commercial NF membranes (DL (Suez (GE)^TM^), NFW NF membrane (Synder^TM^), and NDX (Synder^TM^)) were used during the consecutive fractionation trials. Each treatment was carried out separately and apart from the other treatments (Figure 2). Specific technical properties of these NF membranes are described in Table S1. Two pressure gauges (manometers) were connected to the inside and outside tubbing (SS-316) of the membrane separation cell with the aim of controlling the desired transmembrane pressure (TMP) value accurately. As the outlet for permeation was opened to the air (Figure 2), the average value of these two pressure meters was assumed to be the TMP.

First, three liters of the prepared Muérdago fruit juice was disposed into the system feed tank at room temperature (≈20 °C) and was treated using a DL membrane (DL treatment) (Figure 2) at a TMP of 10 bar and a constant crossflow velocity of 2.85 (L/min) for three hours. The final volumes obtained of both the permeate and the feed solutions were recounted. Then, the feed solution obtained from the DL treatments was reconstituted with distilled water until reaching again a volume of three liters. This reconstituted fruit juice was processed using an NFW membrane (NFW treatment) (Figure 2) at a TMP of 30 bar and a constant crossflow velocity of 2.85 (L/min) for three hours. The final volumes obtained of both the feed and permeate solutions were recounted, and the resultant feed solution (obtained from the NFW treatment) was newly reconstituted with distilled water (pH = 6.5 ± 0.2) until reaching a three-liter volume. Immediately afterwards, this reconstituted solution was processed using an NDX membrane (NDX treatment) (Figure 2) at a TMP of 25 bar and a constant crossflow velocity of 2.85 (L/min) for three hours. Finally, the resultant volumes of the permeate and feed solutions were recounted. Samples of 1.5 mL were taken along each processing trial, at the processing times 5 and 180 min, from the feed and the permeate streams. All the taken samples were immediately refrigerated and kept at 4 °C until rapid analysis. All the fractionation experiments were carried out in a concentration mode. After each membrane treatment, the respective obtained permeate volumes (Figure 2) were stored and not reused during the next consecutive treatments.

The high TMP values used with the membranes NFW and NDX (30 bar and 25 bar, respectively) were chosen according to previous preliminary tests and also because of these membranes having reported to perform better at high TMPs, even close to the membranes’ burst pressures (41 bar) [34]. The concentration of the treated Muérdago extract was kept low in solution (10 g of dry Muérdago powder initially dissolved into 3 L of distilled water). This would allow to observe the fractionation process more clearly, even while membrane fouling appears (forming not excessively thick layers), and the impact on the migration rates and the permeate flux. Membrane fouling by phenolics has been previously observed [35].

The parameters monitored during the fractionation process were solution pH, solution electrical conductivity, and temperature. These parameters helped to understand the mass transfer process and were repeatedly measured during NF [36,37]. The solution was recirculated within a closed stainless steel 316 system, and the permeate was continuously collected into a 250 mL test tube in order to determine the permeate flux along the processing. In addition, each run was carried out in triplicate for all the different treatments realized, and the average value of each parameter measured was a final result. Afterwards, the fouled membranes were evaluated in relation to the permeate flux achieved and then cleaned by washing them up with a cleaning solution of 1% Ultrasil 11 (membrane alkaline detergent) (Henkel, Ecolab, Saint Paul, MN, USA) (pH = 12.0) for at least 1 h, at a slightly elevated temperature (around 40 °C), and at a TMP of 3.5 bar. Finally, the whole system (CF042D cell, pump, SS-316 tubbing) was rinsed several times with deionized water (EC < 4 µS/cm) until the total Ultrasil 11 was removed from the circuit. A test measuring pH and electrical conductivity was then performed in the rinsing water in order to corroborate a clean membrane circuit. Then, the permeate flux of distilled water was determined with the clean membrane material.

### 3.4. Membrane Filtration Assessment

The filtration assessment was performed on the new, the used, and the chemically washed membranes. After the membrane compaction pretreatments, trials using deionized water were carried out on each membrane sample. This was performed before the treatments of the Muérdago fruit solutions and in order to have a record of their respective filtration performances as new membrane material. These trials were also carried out on the fouled membranes after cleaning them with Ultrasil 11. This procedure assessed the effect of fouling formation on the membrane material integrity and the respective performances after the chemical cleaning was realized. The treatments were realized using a crossflow velocity of 2.85 (L/min), at 20 °C, and using TMP values of 5, 10, 15, 20, and 25 bar. At each tested TMP value, the permeate flux was recorded in triplicate considering a filtrated volume of 10 mL for each time-lapse measurement. The TMP value increased slowly and gradually when carrying out the mentioned tests and starting from the lowest up to the highest TMP value, keeping constant each of them while registering the time elapsed. The membrane resistances were calculated according to Equations (1) [38] and (2) [20], considering the slope of each generated curve and the viscosity of water at 20 °C (0.001 Pa*s). The *R_M_* was calculated on the new and on the chemically washed membranes after fouling. This parameter was determined on all the tested membranes (DL, NFW, and NDX). Equation (1) is presented as follows:

(1) $$J=\frac{TMP-∆\pi }{\mu \left(R_{M}+R_{F}+R_{CP}\right)}$$

where *J* is the permeate flux, *TMP* is the transmembrane pressure, *R_M_* is the membrane resistance, *R_F_* is the fouling resistance, *R_CP_* is the concentration polarization resistance, *µ* is the viscosity, and Δ*π* is the osmotic pressure. When the solution is pure water, as in the case of this study, *R_F_* and *R_CP_* become zero and only *R_M_* exists. In that case, *R_M_* can be obtained according to Darcy’s law, as shown in Equation (2):

(2) $$J=\frac{∆P}{\mu R_{M}}$$

where *J* is the permeate flux of sample solution during NF processing (m^3^*m^−2^s^−1^), Δ*P* is the TMP (bar), *μ* is the solution viscosity (bar*s), and *R_M_* is the hydraulic membrane resistance (m^−1^). The membrane resistances were determined for the new, the fouled, and the washed (Ultrasil 11 washing) membranes (DL, NFW, and NDX). The hydraulic MR indicates the membrane integrity and performance.

### 3.5. Retention Percentage (RP)

A particular process variable used to evaluate the membrane fractionation was the retention percentage (*RP*). The *RP* allowed to observe the selective passage of some of the studied molecules through the tested DL, NFW, and NDX membranes. The *RP* was calculated using Equation (3) [39]:

(3) $$RP=\left[1-\left(\frac{C_{P}}{C_{F}}\right)\right]\times 100$$

where *C_P_* and *C_F_* represent the respective solute concentrations (mg/L or ppm) in the permeate and the feed streams, respectively, at a determined processing time.

### 3.6. Permeation Percentage (PP) of Total Solids

The permeation percentage of total solids was determined during each of the treatments realized (for the membranes DL, NFW, NDX) at the processing times 5 and 180 min. It can be defined as the recovery of total compounds in the permeate during the concentration process [40] and was calculated using Equation (4):

(4) $$PP=\frac{C_{p}}{C_{F}}\times 100$$

### 3.7. Electrical Conductivity, pH, and Temperature Measurements

Measurements of electrical conductivity, pH, and temperature were made in the feed, the concentrate, and the permeate streams, during all the treatments realized, using an HI 991,301 pH/EC/TDS/temperature meter (Hanna Instruments, Cluj-Napoca, Cluj, Romania).

### 3.8. Visual Membrane Inspection and Characterization

Digital camera photographs were taken in order to identify and to characterize the aspect of the original membrane material and to compare it with the presence of fouling layers on each of the used membranes DL, NFW, and NDX. This was made in order to visually inspect the active surfaces of the original and of the used active layers of every membrane used and to the detect the presence of membrane fouling in each case. Membrane fouling disturbs the process performance and membrane lifetime.

### 3.9. Tentative Identification (ESI-MS/MS) and Quantification (UHPLC-ESI-MS/MS) of Metabolites in the Muérdago Fruit

Liquid samples of processed Muérdago extract obtained from the feed and from the permeate streams, at the processing times 5 and 180 min, respectively, were filtered and injected in the UHPLC-ESI-MS/MS equipment. Nylon filters (Iso-disc 0.45 µm; Millex-HN, Millex^®^, Merck KGaA, Darmstadt, Germany) were used to filter the final extract before injection. All the samples were analyzed by UHPLC-MS/MS in an Ekspert UltraLC 100-XL ultra-high-pressure liquid chromatograph coupled to an electrospray (ESI) ABSciex Triple Quad 4500 triple quadrupole mass spectrometer. A PhenomenexSynergi™ Fusion-RP 80 Å (50 mm × 2.0 mm, 4 μm) column was employed, and the mobile phase was prepared from 0.1% *v*/*v* formic acid in water (eluent A) and acetonitrile (eluent B). All the constituents of the mobile phases were HPLC-grade. The gradient was programmed as follows: (time, min/%B) 1/5%, 12/50%, 13/50%, 14/5%, and 15/5%. The mass spectrometer parameters were gas 1 N_2_ (40 psi); gas 2 N_2_ (50 psi); ion spray voltage, 3500 V; ion source temperature, 650 °C; curtain gas N_2_ (25 psi); flow 0.3 mL/min; and scan mode MRM with both positive and negative polarity. The UHPLC-MS/MS system was controlled with Analyst 1.6.2, and the data were processed with Multiquant 3.0. Calibration curves were built for each compound in the 0.1–0.8 μg/mL range. The high resolution and accurate mass via orbitrap (HESI orbitrap HR-MS) used in this study enabled the identification and tentative characterization of compounds including phenolics and amino acids. Some of the identified phenolics from all the detected ones in the extract were directly identified, without using references, and quantified by UHPLC-MS, which are presented later on.

### 3.10. Statistical Analysis

Data obtained were subjected to one-way ANOVA using software Sigmaplot (Sigmaplot 14.0, Systat Software Inc. San Jose, CA, USA) in order to compare the mean values of the calculated membrane resistances and some other parameters. Two-way ANOVA was used to compare the mean values of the calculated permeation percentages of the total solids in solution. Two-way repeated measures (RMs) ANOVA was used to evaluate the evolution of certain parameters measured in the solution samples through the processing time and the influence of two independent variables on the obtained data. All the experiments were carried out in triplicate. The power of all the performed tests was the standard criteria for significance (α = 0.0500). Values of *p* < 0.0500 were considered as denoting a significant statistical difference among average parameter values.

## 4. Conclusions

The protocol realized and the membranes tested allowed an interesting fractionation of bioactive compounds that are present in the Muérdago fruit and generate considerable permeate flux. Some phenolics were highly retained by the membranes tested, while some amino acids permeated them progressively and selectively (aspartic acid, proline, tryptophan, valine, leucine, isoleucine, methionine, arginine), as the solution was continuously reconstituted. Tryptophan was found only in the DL permeate fractions. These DL permeate fractions contained also the phenolics quinic acid, 3-CQA, cryptochlorogenic acid, chlorogenic acid, and p-coumaric acid. On the other hand, whereas leucine and isoleucine permeated only the membrane NFW, the amino acids methionine and arginine were found only in the NDX permeate fractions. The NFW permeates contain the phenolics quinic acid, 3-CQA, ferulic acid, cryptochlorogenic acid, chlorogenic acid, and coumaric acid. The NDX permeates contain mainly the phenolics 3-CQA and ferulic acid.

Membrane fouling was observed during each membrane treatment carried out, but it was successfully removed from each membrane after a chemical cleaning treatment and recovered the initial performance. It was observed that the DL membrane allows higher permeate flux at lower TMP values than the NFW and the NDX ones. The DL membrane also allows the more important permeation amounts of total compounds, followed by the NFW membrane and then by the NDX membrane. Permeate fractions of great interest to the food and pharmaceutical industries were obtained and are suitable for process optimization and scale-up. Membrane technology shows promising applicability for the extraction of metabolites from numerous fruit and vegetable juices. Nevertheless, each different process must be carefully studied and optimized in terms of performance and membrane fouling formation.

## Figures and Tables

**Table 1:** Permeate flux observed during the membrane treatments realized.

Membrane Treatments and Permeate Flux (L/m^2^h)			
Proc. Time (min)	DL	NFW	NDX
5	27.73 ± 0.52 ^a^	12.40 ± 0.18 ^c^	16.44 ± 0.39 ^d^
180	22.10 ± 1.26 ^b^	14.45 ± 0.53 ^c^	20.82 ± 0.23 ^e^

Means within columns and rows followed by different letters are significantly different (*p* < 0.0500).

**Table 2:** Membrane resistance values observed for the different membranes tested (original membranes and those used and washed chemically).

Membrane Treatments and Membrane Resistance (m^−1^)			
Membrane Treatment	DL Membrane	NFW Membrane	NDX Membrane
**New membrane**	3.42 × 10^13^ ± 8.77 × 10^11 a^	2.51 × 10^14^ ± 1.01 × 10^12 b^	2.45 × 10^14^ ± 4.61 × 10^12 c^
**Washed membrane**	3.42 × 10^13^ ± 1.71 × 10^11 a^	2.51 × 10^14^ ± 4.11 × 10^12 b^	2.41 × 10^14^ ± 2.90 × 10^12 c^

Means within columns and rows followed by different letters are significantly different (*p* < 0.050).

**Table 3:** Processing parameters measured in the feed streams during the membrane treatments DL, NFW, and NDX (electrical conductivity and pH value).

Membrane Treatments and Processing Parameters			
**Electrical Conductivity (mS/cm)**			
**Processing Time (min)**	**DL**	**NFW**	**NDX**
0	0.36 ± 0.01 ^e^	0.36 ± 0.01 ^i^	0.35 ± 0.01 ^il^
5	0.38 ± 0.01 ^de^	0.37 ± 0.01 ^hi^	0.35 ± 0.01 ^hkl^
60	0.39 ± 0.01 ^c^	0.37 ± 0.01 ^g^	0.36 ± 0.01 ^gk^
120	0.41 ± 0.01 ^b^	0.38 ± 0.01 ^f^	0.38 ± 0.01 ^fj^
180	0.43 ± 0.01 ^a^	0.39 ± 0.01 ^f^	0.39 ± 0.01 ^fj^
**pH value**			
**Processing Time (min)**	**DL**	**NFW**	**NDX**
0	5.99 ± 0.01 ^a^	5.02 ± 0.02 ^f^	4.63 ± 0.02 ^k^
5	5.96 ± 0.02 ^b^	4.99 ± 0.02 ^g^	4.58 ± 0.01 ^l^
60	5.92 ± 0.01 ^c^	4.93 ± 0.01 ^h^	4.55 ± 0.02 ^m^
120	5.90 ± 0.02 ^d^	4.87 ± 0.02 ^i^	4.46 ± 0.01 ^n^
180	5.85 ± 0.01 ^e^	4.81 ± 0.02 ^j^	4.44 ± 0.02 ^o^

Means within a column or row followed by different letters are significantly different (*p* < 0.0500).

**Table 4:** Concentrations of the phenolics quantified through the UHPLC-MS analysis in the feed and in the permeate samples during the treatments DL, NFW, and NDX at the processing time 180 min.

**Phenolic**	**Retention Time (min)**	**Feed DL (ppb)**	**Permeate DL (ppb)**	**Feed NFW (ppb)**
**Gallic acid**	1.24	39.16 ± 0.31 ^ae^	0.00 ± 0.00	41.26 ± 3.31 ^ad^
**Cryptochlorogenic acid**	3.57	5,447,927.57 ± 4,314,977.29 ^af^	25,629.66 ± 25,629.66 ^bc^	5,647,777.10 ± 4,304,459.83 ^ae^
**Chlorogenic acid**	3.94	13,138.29 ± 541.33 ^ag^	11.16 ± 0.58 ^bd^	16,292.64 ± 965.49 ^cf^
**Caffeic acid**	4.29	84.48 ± 4.77 ^ah^	0.00 ± 0.00	181.60 ± 12.30 ^bg^
**p-coumaric acid**	5.48	33.33 ± 0.49 ^ai^	2.02 ± 1.05 ^be^	39.64 ± 1.96 ^cd^
**Rutin**	5.84	124.82 ± 36.85 ^ah^	0.00 ± 0.00	62.84 ± 44.25 ^ad^
**Quercetin**	8.26	265.15 ± 100.36 ^ah^	0.00 ± 0.00	61.32 ± 3.29 ^bd^
**Phenolic**	**Retention time (min)**	**Permeate NFW (ppb)**	**Feed NDX (ppb)**	**Permeate NDX (ppb)**
**Gallic acid**	1.24	0.00 ± 0.00	39.53 ± 33.77 ^ae^	0.00 ± 0.00
**Cryptochlorogenic acid**	3.57	134,035.57 ± 78,074.49 ^cd^	5,633,352.06 ± 4,244,817.36 ^af^	273,929.31 ± 136,748.71 ^cf^
**Chlorogenic acid**	3.94	129.41 ± 1.35 ^de^	16,056.81 ± 846.08 ^cg^	216.91 ± 11.48 ^eg^
**Caffeic acid**	4.29	0.00 ± 0.00	264.06 ± 10.14 ^ch^	0.00 ± 0.00
**p-coumaric acid**	5.48	38.37 ± 0.84 ^cf^	32.20 ± 0.79 ^de^	15.46 ± 0.11 ^eh^
**Rutin**	5.84	0.00 ± 0.00	71.16 ± 69.42 ^ae^	0.00 ± 0.00
**Quercetin**	8.26	0.00 ± 0.00	73.46 ± 6.13 ^ce^	0.00 ± 0.00

Means within a row or column followed by different letters are significantly different (*p* < 0.0500).

**Table 5:** Rejection percentages observed for the quantified phenolics during the three membrane treatments carried out (DL, NFW, NDX) at the processing time 180 min.

Phenolics/Membranes	Rejection Percentage (%)		
	DL	NFW	NDX
**Gallic acid**	100 ± 0.00 ^a^	100 ± 0.00 ^a^	100 ± 0.00 ^a^
**Cryptochlorogenic acid**	99.74 ± 0.21 ^b^	96.85 ± 0.83 ^d^	92.98 ± 2.34 ^g^
**Chlorogenic acid**	99.91 ± 0.01 ^b^	99.20 ± 0.05 ^e^	98.65 ± 0.00 ^h^
**Caffeic acid**	100 ± 0.00 ^a^	100 ± 0.00 ^a^	100 ± 0.00 ^a^
**p-coumaric acid**	84.51 ± 6.43 ^c^	2.64 ± 2.21 ^f^	51.95 ± 1.23 ^i^
**Rutin**	100 ± 0.00 ^a^	100 ± 0.00 ^a^	100 ± 0.00 ^a^
**Quercetin**	100 ± 0.00 ^a^	100 ± 0.00 ^a^	100 ± 0.00 ^a^

Means within a column or a row in the table followed by different letters are significantly different (*p* < 0.0500).

**Table 6:** Permeation percentages (PPs) observed on the membrane cell for each membrane tested at the processing times 5 and 180 min.

Membrane Treatment (Processing Time)	PP (%)
**DL(t5)**	20.63 ± 0.13 ^a^
**DL(t180)**	18.02 ± 0.18 ^b^
**NFW(t5)**	9.89 ± 0.13 ^f^
**NFW(t180)**	11.13 ± 0.11 ^e^
**NDX(t5)**	12.54 ± 0.21 ^d^
**NDX(t180)**	15.49 ± 0.08 ^c^

Mean values within a column followed by different letters are significantly different (*p* < 0.0500).

## Data Availability

The data that support the findings of this study are available from the corresponding author upon reasonable request.

## Supplementary Materials

The following supporting information can be downloaded at https://www.mdpi.com/article/10.3390/plants13111521/s1, Table S1: Technical specifications for the NF membranes DL (Suez (GE)^TM^), NFW (Synder^TM^), and NDX (Synder^TM^), Table S2: List of the metabolites that were identified tentatively ([[M + H]^−^, M + H]^+^) and those quantified by UHPLC-ESI-MS/MS during the membrane treatments carried out (DL, NFW, and NDX), Figure S1: Curves of TMP (bar) values versus permeate flux (L/m^2^h) for the new membranes tested during the filtration of distilled water: (a) DL membrane, (b) NFW membrane, and (c) NDX membrane, (processing temperature: 20 °C), and Figure S2: Schematic membrane fractionation process and representation of the identified molecules in each stream (feed and permeate) during the treatments DL, NFW, and NDX, respectively. The mentioned membrane treatments were carried out consecutively and separately.
