# Supplementary material for: Green Separation by Using Nanofiltration of Tristerix tetrandus Fruits and Identification of Its Bioactive Molecules through MS/MS Spectrometry
Source: Plants (Basel). 2024 May 31;13(11):1521. doi: 10.3390/plants13111521 (PMC11175056; doi:10.3390/plants13111521)
Supplement: Supplementary file 1 [file plants-13-01521-s001.zip › plants-2926673-supplementary.pdf]

Supplementary Material

Green separation by using nanofiltration of *Tristerix tetrandus* fruits and identification of its bioactive molecules through MS/MS spectrometry

Nicolás Cifuentes-Araya[1], Mario Simirgiotis[2], Beatriz Sepúlveda[3],* and Carlos Areche[1,*]

[1] Departamento de Química, Facultad de Ciencias, Universidad de Chile, Las Palmeras 3425, Nuñoa, Santiago, Chile ; areche@uchile.cl

[2]Instituto de Farmacia, Facultad de Ciencias, Campus Isla Teja, Universidad Austral de Chile, Valdivia 5090000, Chile

[3] Departamento de Ciencias Químicas, Universidad Andrés Bello, Campus Viña del Mar, Quillota 980, Viña del Mar 2520000, Chile; bsepulveda@uc.cl

* Correspondence: areche@uchile.cl, (C. Areche), bsepulveda@uc.cl (B. Sepulveda)

Membrane material specification

Table S1. Technical specifications for the NF membranes DL (Suez (GE)^{TM}), NFW (Synder^{TM}), and NDX (Synder^{TM}).

Series	DL	NFW	NDX
**Feed**	Foods/Industrial	Foods/Industrial/Wastewater	Foods/Industrial/Wastewater
**Type**	Low Energy, Low Pressure	Softening	High rejection, Softening
**pH Range**	2-10	4-10	3-10.5
**Flux (GFD)/psi**	28/220	50-55/110	35-45/110
**$MgSO_4$ Rejection**	98.0%	97.0%	95.0%
**Pore size/MWCO**	~150-300 Da	~300-500 Da	~500-700 Da
**Polymer**	Polyamide-TFC	Polyamide-TFC	Polyamide-TFC

* Information provided by Sterlitech Corp. (USA).

Table S2. List of the metabolites that were identified tentatively ([[M+H]$^-$, M+H]$^+$), and those quantified by UHPLC-ESI-MS-MS during the membrane treatments carried-out (DL, NFW, and NDX). The table also shows some particular molecular and chemical properties of the presented metabolites.

Tentative Identification	Molecular weight (Da)	Theoretical Mass (*m/z*)	[M]	[M-H]$^-$	[M+H]$^+$	F-DL(t5)	P-DL(t180)	F-NFW(t5)	P-NFW(t180)	F-NDX(t5)	P-NDX(t180)	pKa	References
							**Phenolics**						
Quinic acid	192.17	192.06	$C_7H_{12}O_6$	$C_7H_{11}O_6^-$	$C_7H_{13}O_6^+$	Yes	Yes	Yes	Yes	Yes		3.46	Simirgiotis et al. 2016, Yang and Rainville, 2020
3-O-caffeoylquinic acid (3-CQA)	354.31	354.10	$C_{16}H_{18}O_9$	$C_{16}H_{17}O_9^-$	$C_{16}H_{19}O_9^+$	Yes	Yes	Yes	Yes	Yes	Yes	3.59, 8.59	Simirgiotis et al. 2016, Fernández-Galleguillos et al. 2023
p-coumaroyl malate	280.23	280.06	$C_{13}H_{12}O_7$	$C_{13}H_{11}O_7^-$	$C_{13}H_{13}O_7^+$	Yes				Yes		3.33	Simirgiotis et al. 2016
Isorhamnetin	315.02	316.06	$C_{16}H_{12}O_7$	$C_{16}H_{11}O_7^-$	$C_{16}H_{13}O_7^+$						Yes	6.38	Simirgiotis et al. 2016, Fernández-Galleguillos et al. 2023
Apigenin	269.05	270.05	$C_{15}H_{10}O_5$	$C_{15}H_9O_5^-$	$C_{15}H_{11}O_5^+$					Yes		6.57	de Dicastillo et al. 2017, Simirgiotis et al. 2016
Ferulic acid	194.18	194.06	$C_{10}H_{10}O_4$	$C_{10}H_9O_4^-$	$C_{10}H_{11}O_4^+$			Yes	Yes		Yes	4.30, 8.81	Li et al. 2018, Cabrera-Barjas et al. 2020
Ellagic acid	302.20	302.01	$C_{14}H_6O_8$	$C_{14}H_5O_8^-$	$C_{14}H_7O_8^+$	Yes		Yes		Yes		6.69, 7.45, 9.61, 11.50	Jincy and Sunil, 2022, de Dicastillo et al. 2017
7-O-Methylisorhamnetin	329.07	329.07	$C_{17}H_{14}O_7$	$C_{17}H_{13}O_7^-$	$C_{17}H_{15}O_7^+$						Yes		Simirgiotis et al. 2016S
**Compounds identified and quantified**								**Phenolics**					
Gallic acid	170.12	170.02	$C_7H_6O_5$	$C_7H_5O_5^-$	$C_7H_7O_5^+$	Yes		Yes		Yes		4.4, 8.5, 10.3, 13.0	López et al. 2018, Junqueira-Gonçalves et al. 2015, Pérez-Almeida et a. 2022
Cryptochlorogenic acid	354.31	354.10	$C_{16}H_{18}O_9$	$C_{16}H_{17}O_9^-$	$C_{16}H_{19}O_9^+$	Yes	Yes	Yes	Yes	Yes	Yes	3.44	Simirgiotis et al. 2016
Chlorogenic acid	354.31	354.10	$C_{16}H_{18}O_9$	$C_{16}H_{17}O_9^-$	$C_{16}H_{19}O_9^+$	Yes	Yes	Yes	Yes	Yes	Yes	3.59, 8.59	Simirgiotis et al. 2016, Navarro-Orcajada et al. 2021, Fernández-Galleguillos et al. 2023
Caffeic acid	180.16	180.04	$C_9H_8O_4$	$C_9H_7O_4^-$	$C_9H_9O_4^+$	Yes		Yes		Yes		4.62, 9.07, 11.2	López et al. 2018, Salehi et al. 2021, Navarro-Orcajada et al. 2021
p-Coumaric acid	164.16	164.05	$C_9H_8O_3$	$C_9H_7O_3^-$	$C_9H_9O_3^+$	Yes	Yes	Yes	Yes	Yes	Yes	4.34, 8.83	Junqueira-Gonçalves et al. 2015, Aguilar-Hernández et al. 2017
Rutin	610.52	610.15	$C_{27}H_{30}O_{16}$	$C_{27}H_{29}O_{16}^-$	$C_{27}H_{31}O_{16}^+$	Yes		Yes		Yes		7.21, 7.52	Xiao et al. 2021
Quercetin	302.23	302.40	$C_{15}H_{10}O_7$	$C_{15}H_9O_7^-$	$C_{15}H_{11}O_7^+$	Yes		Yes		Yes		7.17, 8.26, 10.13, 12.30, 13.11	Junqueira-Gonçalves et al. 2015, Aguilar-Hernández et al. 2017, Xiao et al. 2021

Amino acids													
**Tentative Identification**	**Molecular weight (Da)**	**Theoretical Mass (*m*/*z*)**	**[M]**	**$[M-H]^-$**	**$[M+H]^+$**	**F-DL(t5)**	**P-DL(t180)**	**F-NFW(t5)**	**P-NFW(t180)**	**F-NDX(t5)**	**P-NDX(t180)**	**pka1 (α-carboxyl group), pka2 (α-amino group)**	**References**
Aspartic acid	133.10	133.04	$C_4H_7NO_4$	$C_4H_6O_4N^-$	$C_4H_8O_4N^+$	Yes	Yes	Yes	Yes	Yes		1.88, 3.65, 9.60	Cabrera-Barjas et al. 2020, Idrees et al. 2020
Proline	115.13	115.06	$C_5H_9NO_2$	$C_5H_8O_2N^-$	$C_5H_{10}O_2N^+$		Yes		Yes			1.99, 10.60	Cabrera-Barjas et al. 2020, Idrees et al. 2021
Valine	117.15	117.08	$C_5H_{11}NO_2$	$C_5H_{10}O_2N^-$	$C_5H_{12}O_2N^+$			Yes	Yes	Yes	Yes	2.32, 9.62	Cabrera-Barjas et al. 2020, Idrees et al. 2022
Tryptophan	204.23	204.09	$C_{11}H_{12}N_2O_2$	$C_{11}H_{11}N_2O_2^-$	$C_{11}H_{12}N_2O_2^+$		Yes					2.83, 9.39	Cabrera-Barjas et al. 2020, Idrees et al. 2023
Leucine	131.17	131.17	$C_6H_{13}NO_2$	$C_6H_{12}NO_2^-$	$C_6H_{14}NO_2^+$				Yes			2.36, 9.60	Cabrera-Barjas et al. 2020, Idrees et al. 2024
Isoleucine	131.17	131.17	$C_6H_{13}NO_2$	$C_6H_{12}NO_2^-$	$C_6H_{14}NO_2^+$				Yes			2.36, 9.61	Cabrera-Barjas et al. 2020, Idrees et al. 2025
Methionine	149.21	149.05	$C_5H_{11}NO_2S$	$C_5H_{10}NO_2S^-$	$C_5H_{12}NO_2S^+$						Yes	2.28, 9.21	Cabrera-Barjas et al. 2020, Idrees et al. 2026
Arginine	174.20	172.22	$C_6H_{14}NO_4S$	$C_6H_{13}NO_4S^-$	$C_6H_{15}NO_4S^+$						Yes	2.17, 9.04, 12.48	Cabrera-Barjas et al. 2020, Idrees et al. 2027

Membrane permeate filtration

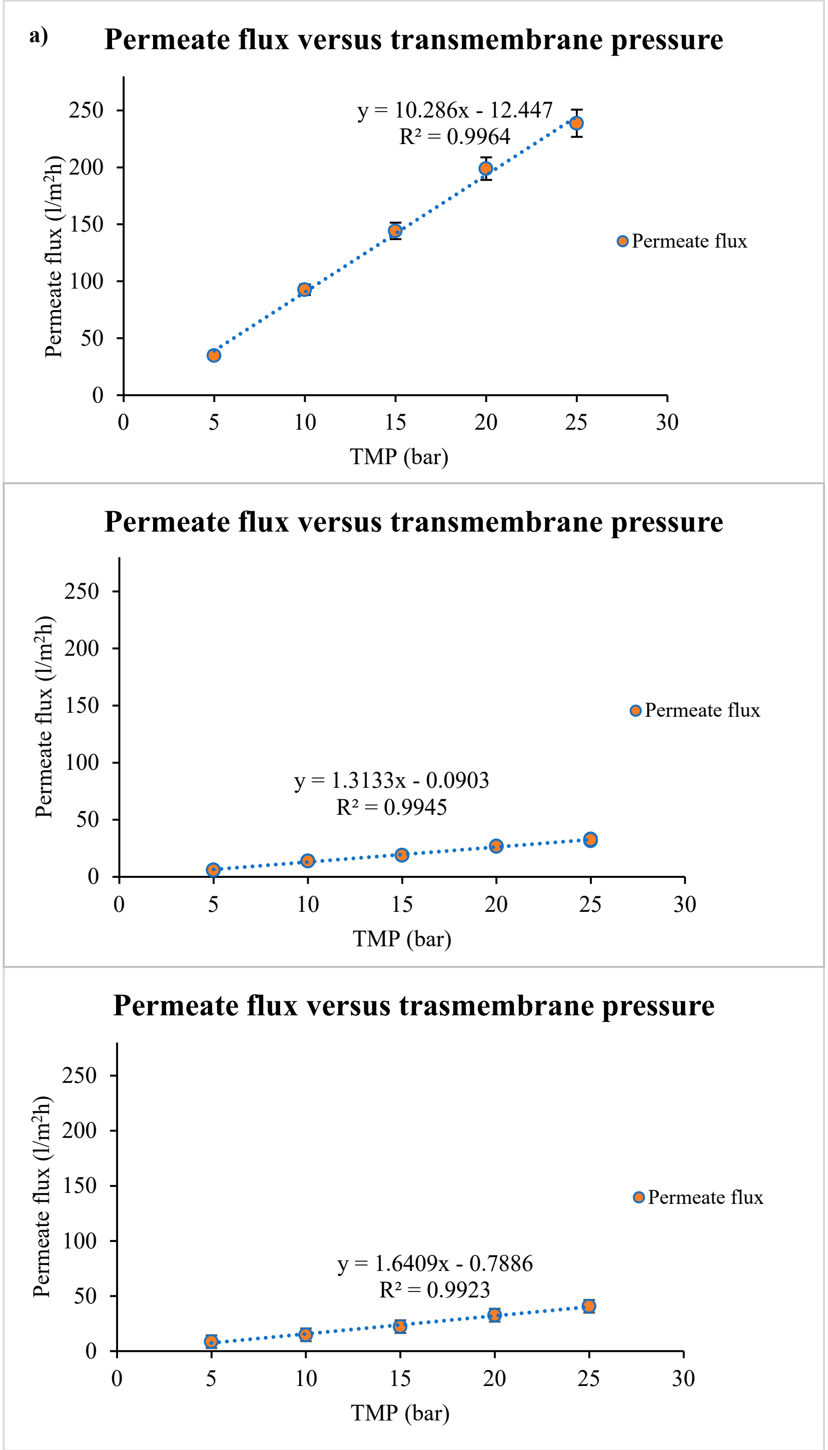

Figure S1. Curves of TMP (bar) values versus permeate-flux (l/m^2h) for the new membranes tested during the filtration of distilled water: a) DL membrane, b) NFW membrane, c) NDX membrane, (processing temperature: 20 °C).

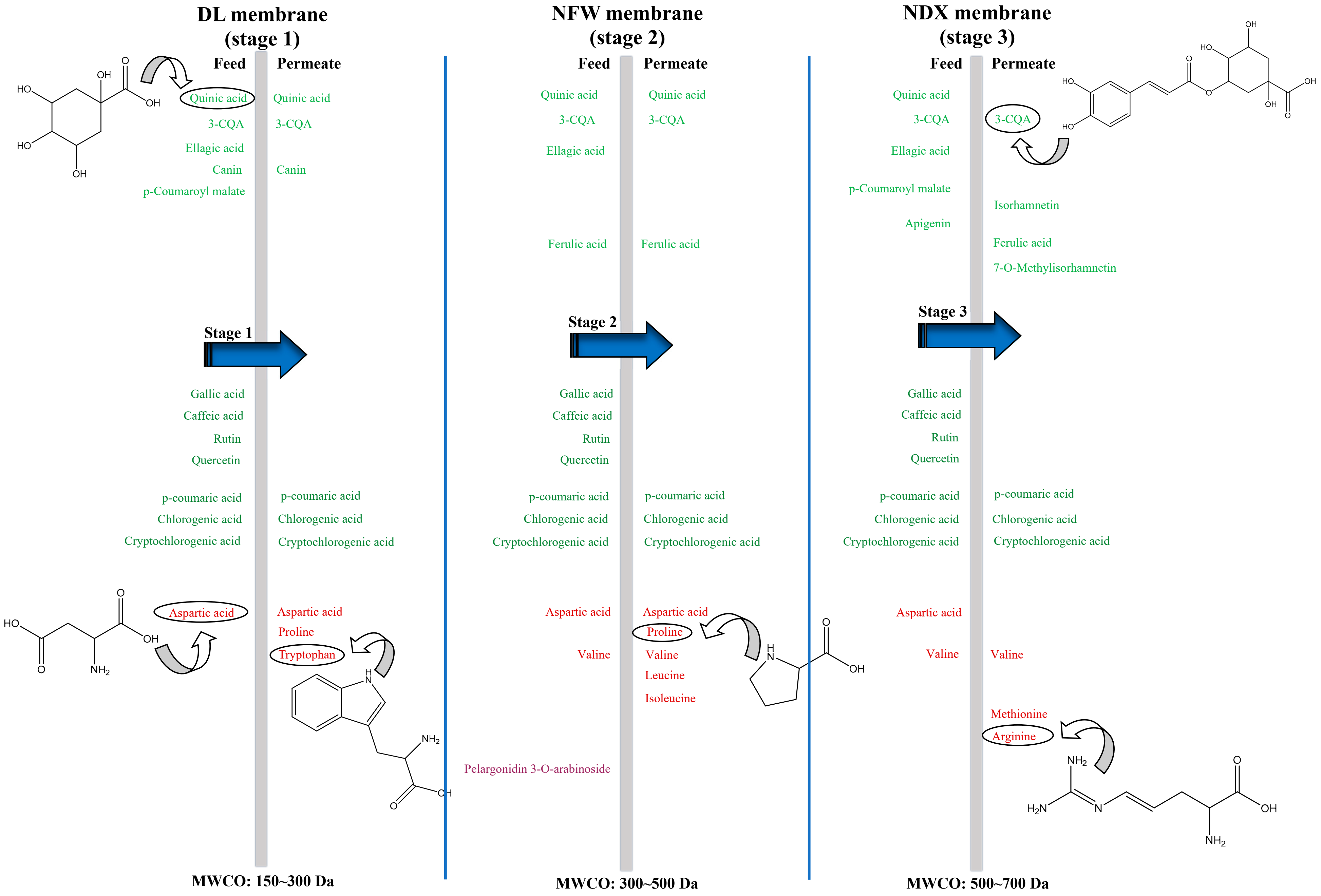

DL membrane
(stage 1)
Feed
Permeate
Quinic acid
Quinic acid
3-CQA
3-CQA
Ellagic acid
Canin
Canin
p-Coumaroyl malate
Stage 1
Gallic acid
Caffeic acid
Rutin
Quercetin
p-coumaric acid
p-coumaric acid
Chlorogenic acid
Chlorogenic acid
Cryptochlorogenic acid
Cryptochlorogenic acid
Aspartic acid
Aspartic acid
Proline
Tryptophan
MWCO: 150~300 Da
NFW membrane
(stage 2)
Feed
Permeate
Quinic acid
Quinic acid
3-CQA
3-CQA
Ellagic acid
Ferulic acid
Ferulic acid
Stage 2
Gallic acid
Caffeic acid
Rutin
Quercetin
p-coumaric acid
p-coumaric acid
Chlorogenic acid
Chlorogenic acid
Cryptochlorogenic acid
Cryptochlorogenic acid
Aspartic acid
Aspartic acid
Proline
Valine
Valine
Leucine
Isoleucine
Pelargonidin 3-O-arabinoside
MWCO: 300~500 Da
NDX membrane
(stage 3)
Feed
Permeate
Quinic acid
3-CQA
3-CQA
Ellagic acid
p-Coumaroyl malate
Isorhamnetin
Apigenin
Ferulic acid
7-O-Methylisorhamnetin
Stage 3
Gallic acid
Caffeic acid
Rutin
Quercetin
p-coumaric acid
p-coumaric acid
Chlorogenic acid
Chlorogenic acid
Cryptochlorogenic acid
Cryptochlorogenic acid
Aspartic acid
Valine
Valine
Methionine
Arginine
MWCO: 500~700 Da

Figure S2. Schematic membrane fractionation process and representation of the identified molecules in each stream (feed and permeate) during the treatments DL, NFW, and NDX, respectively. The mentioned membrane treatments were carried-out consecutively.

Supporting references

1. Simirgiotis, M.J., Quispe, C., Areche, C., Sepúlveda, B. (2016). Phenolic compounds in chilean mistletoe (Quintral, Tristerix tetrandus) analyzed by UHPLC-Q/Orbitrap/MS/MS and its antioxidant properties. Molecules, 21, 245. 10.3390/molecules21030245.

2. Yang, J., Rainville, P. (2020). Analysis of organic acids using a mixed-mode LC column and an ACQUITY QDa mass detector. Waters Technology brief. 720006938EN.

3. Fernández-Galleguillos, C., Jiménez-Aspee, F., Mieres-Castro, D., Rodríguez-Núñez, Y.A., Gutiérrez, M., Guzmán, L., Echeverría, J., Sandoval-Yáñez, C., Forero-Doria, O. (2023). Phenolic profile and cholinesterase inhibitory properties of three chilean altiplano plants: Clinopodium gilliesii (Benth.) Kuntze [Lamiaceae], Mutisia acuminata Ruiz & Pav. var. Hirsuta (Meyen) Cabrera, and Tagetes multiflora (Kunth) [Asteraceae]. Plants, 12, 819. https://doi.org/10.3390/plants12040819.

4. de Dicastillo, C.L., Bustos, F., Valenzuela, X., López-Carballo, G., Vilariño, J.M., Galotto, M.J. (2017). Chilean berry *Ugni molinae* Turcz. fruit and leaves extracts with interesting antioxidant, antimicrobial and tyrosinase inhibitory properties. Food Res. Int., 102, 119-128. 10.1016/j.foodres.2017.09.073.

5. Li, Q., Yang, S., Li, Y., Xue, X., Huang, Y., Luo, H., Zhang, Y., Lu, Z. (2018). Comparative Evaluation of Soluble and Insoluble-Bound Phenolics and Antioxidant Activity of Two Chinese Mistletoes. Molecules, *23*, 359.

6. Jincy, J., Sunil, C. (2020). Exploring antiulcer and anti-inflammatory activities of methanolic leaves extract of an Indian mistletoe *Helicantes elasticus* (Desv.) Danser. South African Journal of Botany, 133, 10-16. https://doi.org/10.1016/j.sajb.2020.06.014.

7. Cabrera-Barjas, G., Quezada, A., Bernardo, Y., Moncada, M., Zúñiga, E., Wilkens, M.., Giordano, A., Nesic, A., Delgado, N. (2020). Chemical composition and antibacterial activity of red murta (Ugni molinae Turcz.) seeds: an undervalued Chilean resource. J. Food Meas. Charact., 14, 1810-1821. https://doi.org/10.1007/s11694-020-00428-x.

8. Alfei, S., Marengo, B., Zuccari, G. (2020). Oxidative Stress, Antioxidant Capabilities, and Bioavailability: Ellagic Acid or Urolithins?. Antioxidants, 9, 707. 10.3390/antiox9080707.

9. López, J., Vega-Gálvez, A., Angela Rodríguez, A., Uribe, E., Bilbao-Sainz, C. (2018). Murta (Ugni molinae Turcz.): A review on chemical composition, functional components and biological activities of leaves and fruits. Chilean J. Agric. Anim. Sci., ex Agro-Ciencia, 34(1), 43-56. http://dx.doi.org/10.4067/S0719-38902018005000205.

10. Junqueira-Gonçalves, M.P., Yáñez, L. Morales, C., Navarro, M., Contreras, R.A., Zúñiga, G.E. (2015). Isolation and characterization of phenolic compounds and anthocyanins from Murta (Ugni molinae Turcz.) fruits. Assessment of Antioxidant and Antibacterial Activity. Molecules, 20, 5698-5713. doi:10.3390/molecules20045698.

11. Pérez-Almeida, N., González, A.G., Santana-Casiano, J.M., González-Dávila, M. (2022). Ocean acidification effect on the iron-gallic acid redox interaction in seawater. Front. Mar. Sci. 9:837363. 10.3389/fmars.2022.837363.

12. Navarro-Orcajada, S., Matencio, A., Vicente-Herrero, C., García-Carmona, F. López-Nicolás, J.M. (2021). Study of the fluorescence and interaction between cyclodextrins and neochlorogenic

acid, in comparison with chlorogenic acid. Scientific Reports, 11(1), 1-11. https://doi.org/10.1038/s41598-021-82915-9.

13. Salehi, B., Sharifi-Rad, J., Herrera-Bravo, J., Salazar, L.A., Delporte, C., Valenzuela-Barra, G., Cazar-Ramírez, M.E., López, M.D., Ramírez-Alarcón, K., Cruz-Martins, N., Martorell, M. (2021). Ethnopharmacology, Phytochemistry and Biological Activities of Native Chilean Plants. Curr. Pharm. Des., 27, 953-970. 10.2174/1381612826666201124105623.

14. Aguilar-Hernández, I., Afseth, N.K., López-Luke, T., Contreras-Torres, F.F., Wold, J.P., Ornelas-Soto. N. (2017). Surface enhanced Raman spectroscopy of phenolic antioxidants: A systematic evaluation of ferulic acid, *p*-coumaric acid, caffeic acid and sinapic acid. Vib. Spectrosc., 89, 113-122, https://doi.org/10.1016/j.vibspec.2017.02.002.

15. Xiao, Z., He, L., Hou, X., Wei, J., Ma, X., Gao, Z., Yuan, Y., Xiao, J., Li, P., Yue, T. (2021). Relationships between Structure and Antioxidant Capacity and Activity of Glycosylated Flavonols. Foods 2021, 10, 849. https://doi.org/10.3390/foods10040849.

References to websites

1. Sterlitech Corporation, Flat Sheet Membranes. https://www.sterlitech.com/flat-sheet-membranes.html/

2. HMDB, The Human Metabolome Database, https://hmdb.ca/

3. Foodb, Listing Compounds, https://foodb.ca/compounds/

4. Drugbank Online, https://go.drugbank.com/drugs/

5. PubChem, National Library of Medicine, https://pubchem.ncbi.nlm.nih.gov/

6. PhytoHub, https://phytohub.eu/
